# Oral cancer stem cells - properties and consequences

**DOI:** 10.1590/1678-7757-2016-0665

**Published:** 2017

**Authors:** Camila Oliveira Rodini, Nathália Martins Lopes, Vanessa Soares Lara, Ian Campbell Mackenzie

**Affiliations:** 1 Universidade de São Paulo, Faculdade de Odontologia de Bauru, Departamento de Ciências Biológicas, Bauru, SP, Brasil.; 2 Universidade de São Paulo, Faculdade de Odontologia de Bauru, Departamento de Cirurgia, Estomatologia, Patologia e Radiologia. Bauru, SP, Brasil.; 3 Queen Mary University of London, Blizard Institute - Barts and The London School of Medicine and Dentistry, London, United Kingdom.

**Keywords:** Neoplastic stem cells, Mouth neoplasms, Epithelial-mesenchymal transition, Neoplasm metastasis, Squamous cell carcinoma

## Abstract

Research on cancer stem cells (CSCs) has greatly increased in the field of medicine and pathology; however, some conceptual misunderstandings are still present among the public as well as within the general scientific community that is not yet familiar with the subject. The very first problem is the misinterpretation of CSCs as a synonym of their normal counterparts, the well-known stem cells (SCs). Particularly in Dentistry, another common mistake is the misinterpretation of oral CSCs as normal tooth-derived SCs. The present review aims to clarify important concepts related to normal SCs and CSCs, as well as discuss the relevance of CSCs to the development, metastasis and therapy resistance of oral squamous cell carcinoma.

## Introduction

For several years the concept of “stem cells” has been of general scientific interest and has raised the important prospect of being able to create new human tissues in the laboratory and use them to replace those lost by injury or disease. Such stem cell concepts applied to Dentistry, as well as to general Medicine, could be greatly enhanced by researches exploring the ability of stem cells to generate new tissues such as mucosa and bone tissues and, eventually, regenerate dental tissues including perhaps even the whole teeth. Recently, however, the dental literature has begun to contain references to “cancer stem cells” (CSCs) and these have quite a different concept. These are the cells that have the ability to stimulate the growth of oral cancers and enable tumours to resist therapy. This review will describe how CSCs differ from normal stem cells, how they can be isolated and studied, how they have special properties and, of most importance, how they are responsible for the spreading of cancer and how they might be targeted for destruction. There is now good evidence that CSCs exist in most tumours but this review will focus mainly on oral squamous cell carcinoma, which comprises the great majority of malignant oral cancers.

### Background of oral cancer

Oral squamous cell carcinoma (OSCC) is the most commonly occurring oral malignancy and one of the most widely occurring cancers throughout the world^9^ ^,^ ^25^ ^,^ ^33^. OSCC is a malignancy that arises in the squamous epithelium lining the oral cavity and includes tumours found on the tongue, lip, gingival, palate, floor of the mouth and buccal mucosa^13^ ^,^ ^25^. The risk factors for development of OSCC include tobacco exposure, alcohol consumption, and infection with oncogenic viruses such as HPV^9^ ^,^ ^35^. The tumour can invade deeply into adjacent tissues of the tongue and and the floor of the mouth, as well as into bones, primarily of the alveolar crest^41^.

Microscopically, OSCC usually shows variable degrees of keratinization, cellular and nuclear pleomorphism, and mitotic activity. They are graded as well-, moderately- or poorly-differentiated (grades 1 to 3) according to WHO criteria^23^ ^,^ ^43^. The tumour's features, including size and site, histologic malignant grade, perineural spread at the invasive front, lymphovascular invasion and tumour thickness, can act as major risk factors influencing the prognosis for OSCC patients^32^; however, the main negative prognostic factor is the presence of lymph node metastasis, which occurs in 25 to 65% of cases^15^ ^,^ ^29^.

The treatment for early-stage OSCC is generally single modality, either surgery or radiotherapy. In cases of locally advanced OSCC, the treatment is multimodal, with either surgery followed by adjuvant radiation or chemo-radiation, as indicated by pathologic features, or definitive chemo-radiation^27^. Approximately half of all patients survive 5 years after treatment and survival is heavily influenced by the stage of the disease at diagnosis^35^.

### The various types of normal stem cells

**Figure 1:**
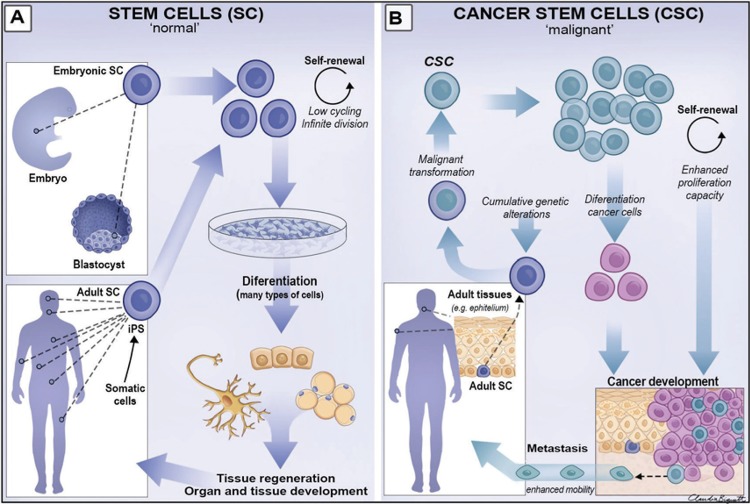
Schematic view of normal stem cells (A) and cancer stem cells (B). A shows different sources of normal SCs, their biological properties of indefinite division through self-renewal and generation of differentiated cells under appropriate conditions; while embryonic stem cells are totipotent, adult stem cells are unipotent but can regain totipotent properties under in vitro conditions, originating the induced pluripotent stem cells (iPSCs). In B, adult epithelial SCs can undergo malignant transformation after cumulative genetic alterations caused by carcinogens, generating CSCs. These CSCs retain the biological properties of the self-renewal and generation of differentiated (cancer) cells, leading to cancer development and further metastasis

The general term “stem cells” includes several different types of cells and the first distinction to be made is between (a) normal stem cells (SC), which are responsible for the development and maintenance of all of the tissues of the body, and (b) their diseased counterpart, called cancer stem cells (CSC), that have lost the close growth control that is a property of normal stem cells. The most primitive type of stem cell is the fertilized egg and its first few divisions early in development produce cells that retain the ability to generate all the different cell types of the adult body. They are therefore described as “totipotent” and, unusually for stem cells, they are transient. As the embryonic development proceeds, these totipotent stem cells become directed towards differentiation into the many distinct tissue types of the adult individual (e.g. stem cells for blood, bones, mucosa, etc.). As they do so they lose some of their developmental potential and become either “pluripotent”, that is, restricted to forming only a few types of tissues, or “unipotent”, restricted to generating only a single tissue. Thus, several subtypes of stem cells exist in adult individuals, each with different potentialities depending on their developmental history. The epithelial stem cells of oral mucosal epithelia are typically unipotent and form only the type of epithelium typical of the region where they are found (Figure 1).

The general property that characterizes adult (somatic) stem cells is that they can be divided indefinitely, normally producing one cell that remains a stem cell and one cell that differentiates itself into a functional tissue cell. This normal “asymmetrical” division pattern is important as it results in the maintenance of the same number of stem cells while also providing another cell for tissue function. However, when it is necessary to replace stem cells, such as those lost after wounding, stem cells can be divided “symmetrically” to form two stem cells and thus increase their number.

### Experimentally derived stem cells

Although normal adult stem cells can divide themselves to regenerate tissues throughout life, they can be difficult to grow for a long period in the laboratory. However, totipotent cells taken early in embryonic development have been shown to continue divide themselves indefinitely in tissue culture without losing their totipotent abilities. For this work, Martin Evans and colleagues were awarded the Nobel Prize in 2007, recognizing the great potential of such “embryonic stem cells” to allow new tissues to be regenerated in the laboratory. Further studies on the genes expressed by embryonic stem cells have identified genes that are responsible for maintaining “sternness” and this led to another major step. It was found that when such genes are artificially expressed in adult stem cells they regain the totipotent properties of embryonic stem cells. For his work with these “induced pluripotent stem cells (iPSCs), Shinya Yamanaka was awarded the Nobel Prize in 2012. These findings are important mainly to the fields of Regenerative Medicine and Tissue Bioengineering. However, this work also shows that although adult stem cells are normally restricted permanently to a particular tissue type, they can be manipulated experimentally to form different cell types (Figure 1). Such cell plasticity is also of interest to cancer development, progression and metastasis. Thus, stem cell research now has two different directions: (a) how to encourage the growth and differentiation of stem cells for tissue regeneration, and (b) how to prevent the growth of cancer stem cells to prevent the expansion, metastasis and recurrence of the tumours in which they are found.

### Normal oral epithelium and oral cancer

Normal oral mucosa is covered by a stratified squamous epithelium and although keratinocytes form the primary cell type of the tissue, these epithelia also contain a minority of cells such as melanocytes, Langerhans cells, Merkel cells, and transient inflammatory cells. Some regions of the mouth have a keratinied or cornified surface layer that is able to resist the forces of mastication, but other regions, where the epithelium acts as a lining that is required to stretch, have a non-keratinied epithelium. Oral epithelia are formed of a number of cell strata known as the basal, spinous, granular and corneal layers in keratinized regions, and as basal, spinous, intermediate and superficial layers in nonkeratinized regions. In all regions, cell proliferation occurs in the basal cell layer to provide new cells that undergo differentiation as they move upwards through the strata and the whole epithelial component of the mucosa is renewed in 5-40 days depending on the region. The rapid tissue renewal that confers a remarkable regenerative potential to the oral mucosa is ultimately related to the presence and dynamics of the epithelial stem cells (eSC) present in the basal cell layer. The carefully ordered structure and balanced cell renewal found in normal oral mucosa is progressively lost with the development of cancer.

**Figure 2:**
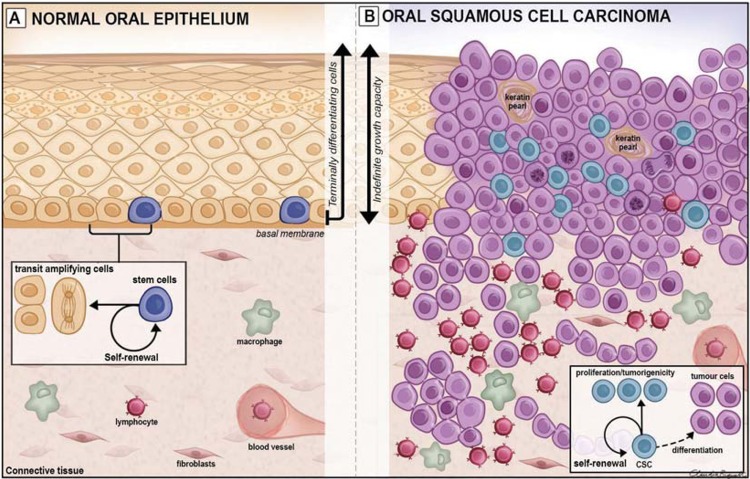
Schematic view of normal oral epithelium (A) and oral squamous cell carcinoma (B). In A, the asymmetric division of normal stem cells (SCs) found in the basal cell layer generates one daughter SC (self-renewal) and one daughter transit-amplifying cell committed to differentiation (small black box). In B, loss of balance of genes related to cell growth and death due to cumulative somatic mutations on SCs results on the development of cancer stem cells (CSCs), which retain the biological properties of self-renewal and generation of differentiated (tumour) cells (small black box). These CSC properties reflect the indefinite growth capacity and morphology heterogeneity of tumours

It was originally thought that all epithelial basal cells are similar and all divide themselves to produce cells that are committed to differentiation as a result of being randomly squeezed out of the basal layer by population pressure. However, measurements of regional differences in proliferation rates, and of the cell lineages produced by labelled cells, indicate that only a small fraction of the proliferating cells have the stem property of indefinite division. The present concept is that such stem cells form only a small fraction of the total dividing cells and that they divide quite slowly to produce cells committed to differentiation. These differentiating “transit-amplifying” (TA) cells have a high proliferative rate but a low self-renewal capacity, so they eventually differentiate into specialized cells that stop dividing and fully differentiate themselves^11^ ^,^ ^12^ (Figure 2).

Such cell proliferation is fundamental for epithelial renewal but it requires tightly controlled mechanisms that balance epithelial cell production and loss. The loss of such balance can be resulted from the increased or decreased expression of genes (proto-oncogenes and their opposite, tumour suppressor genes) related to the control of the cell cycle and death. Accumulation of somatic mutations may alter the expression pattern of genes involved in the control of cell growth and differentiation leading to the loss of proliferative control that characterizes cancer. Such mutations arise as a result of long-term exposure to carcinogens such as tobacco and alcohol (Figures 1B and 2).

### Oral cancer stem cells

As stem cells are ultimately responsible for all the normal tissue growth and renewal occurring in the body, it therefore logically follows that stem cells are also likely to be responsible for cancer growth. However, although the idea that CSCs stimulate the growth and spread of tumours has been discussed for many years, their existence has also been questioned. It might be thought that with the loss of proliferative control and spatial organization, the normal stem cell patterns would disappear. From what has been described above, it can be seen that if all dividing cells of a tumour have equal proliferative abilities they can all be considered stem cells and a sub-population of stem cells would not exist. The experimental difficulty in testing this has been to find good markers for stem cells and to test whether initiation and maintenance of tumours is restricted to the sub-population of the cells identified. The first experimental evidence for the existence of such tumour-initiating stem cells was provided in 1997, when Bonnet & Dick found that only the small subpopulation of leukemic cells, marked by staining, positively for CD34 and negatively for CD38, was able to regenerate the original leukaemia when transplanted into immunodeficient mice^5^. Subsequently, CSCs were identified in solid tumours by Al-Hajj and co-workers who reported in 2003 that only a subpopulation of breast cancer cells staining, positively for CD44 and negatively for CD24, could re­ initiate tumours with the cellular heterogeneity typical of the original tumour^1^. Since then, increasing evidence for the presence of such cells has been found for many tumours including those of the central nervous system, breast, prostate and pancreas^22^ ^,^ ^31^ ^,^ ^39^.

Many evidences that CSCs also play a central role in the pathogenesis and progression of carcinomas of the head and neck (HNSCC), including OSCC, have been found. Early tissue culture studies showed that only a subpopulation of OSCC cells can form expanding tumour colonies, suggesting that human OSCC may contain some form of stem cells^24^ and it was subsequently shown that only a small subpopulation of the cells in OSCC corresponds to tumour-initiating cells^26^ ^,^ ^46^. These findings are in accordance with the CSC concept^17^ ^,^ ^34^ that the tumour mass is a mixture of (a) CSCs dividing themselves to feed the tumour's growth, (b) transient amplifying cells that divide themselves a few times before maturing into (c) differentiated tumour cells that do not contribute to tumour growth^4^.

The isolation of CSCs from oral cancers has mainly been performed with the CD44 marker that was initially used to isolate breast cancer CSCs. CD44 is an adhesion molecule that binds itself to hyaluronan and its expression is necessary for the maintenance of the CSC's properties. CSCs lose their “sternness” when CD44 is experimentally reduced^44^. However, a problem with CD44, and also with all other CSC markers that have been identified so far, is that they are not entirely specific. No single marker is capable of specifically recognizing CSCs and additional markers have therefore been sought. ALDH1 is an intracellular enzyme involved in detoxification and drug resistance via the oxidation of aldehydes, and ALDH-positive cells in HNSCC are reported to have typical CSC behavior and increased tumorigenic ability^21^. The combination of CD44 with other markers, such as ALDH1, may improve the specificity of CSCs' recognition and isolation.

### Cancer stem cells and treatment failure

As they are the cells stimulating tumour growth, elimination of CSCs is necessary for the elimination of tumours. However, many studies have now shown that CSCs are more resistant than other tumour cells to chemotherapy and radiotherapy^7^. *In vitro* assays show that when CD44-high CSCs are irradiated or exposed to chemotherapy, they may be over 10 times more resistant to apoptosis than CD44-low cells^18^. The sensitivity of surrounding normal tissues to high doses of chemo- and radio-therapies restricts the dose levels that can be administered and, despite the various methods of targeting, the dose provided may be sufficient to kill many tumour cells but not all of the CSCs. Clinically, therefore, the tumour may appear to shrink, and even perhaps disappear, only for a few remaining CSCs to begin to divide and subsequently regenerate it. Local tumour recurrence is a major problem for OSCC therapy and elimination of CSCs is a target of therapy but one that is made more complex by the heterogeneity of CSCs as discussed below.

### Epithelial to mesenchymal transition

The epithelial to mesenchymal transition (EMT) was first recognized as a feature of embryogenesis but it is also activated during wound healing and organ fibrosis^40^. Recent evidence indicates that genetic programs relevant for EMT are also activated in epithelial cancers and that the changes induced in cancer cells by EMT appears to play a central role in cancer progression and metastasis^8^ ^,^ ^19^. Epithelial cells are normally attached firmly to the basement membrane and adjacent cells, but EMT allows them to acquire a mesenchymal cell phenotype that is migratory and invasive, and also has elevated resistance to apoptosis^19^ ^,^ ^20^. These changes are characterized by the down-regulation of E-cadherin, translocation of β-catenin from the cell membrane to the nucleus, and up-regulation of mesenchymal molecular markers such as vimentin, fibronectin and N-cadherin^28^ ^,^ ^36^ ^,^ ^42^. There is also up-regulation of transcription factors such as *SNAIL*, *TWIST*, and *LEF-1* that promote EMT^14^ ^,^ ^45^.

**Figure 3:**
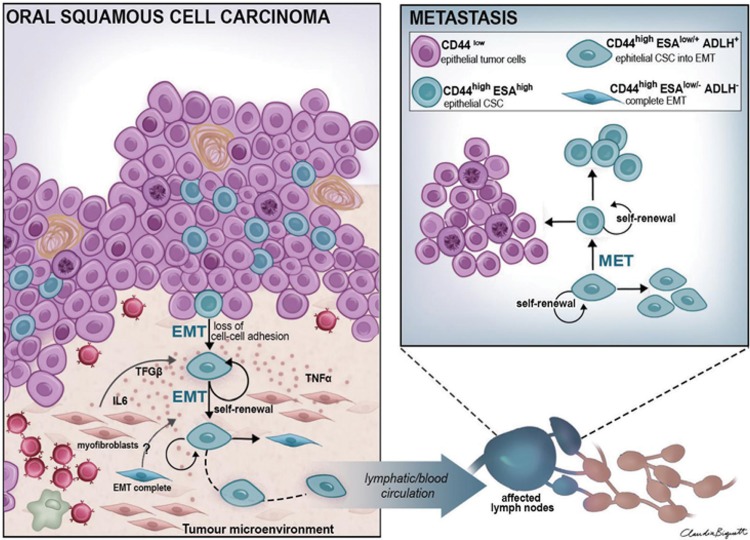
Schematic view of the primary site of oral squamous cell carcinoma (A) and metastatic lymph node (B). In A, cancer stem cells (CSCs) (round blue cells) undergo EMT (EMT CSCs) under the influence of molecules (such as TGF-β, IL6, TNF-α) from the tumour's microenvironment, assuming a complete mesenchymal (CD44^high^ESA^low^/-ALDH-) or epithelial-mesenchymal (CD44^high^ESA^low^/+ALDH+) phenotype. This subpopulation of EMT-CSCs is able to invade the tumour's stroma, migrate and reach blood and lymphatic circulation. Once they arrive at a metastatic lymph node (B), they revert back, through mesenchymal-epithelial transition (MET), to the proliferative non-EMT phenotype (CD44^high^ESAhigh) to enable the formation of a metastatic tumour at that secondary site

Metastasis is a major therapeutic problem for OSCC and the presence of lymph node metastasis is a strong predictor of therapeutic failure. For metastasis of OSCC to occur, cells of the primary tumour need to undergo EMT, invade the surrounding tissue, gain access to lymphatic or blood vessels, and then survive transport to exit from vessels and invade a new tissue site^38^. Through the reverse process of mesenchymal-to-epithelial transition (MET), the cells then transition back to the proliferative epithelial phenotype to form secondary tumours^6^. Applying this to the cancer stem cell concept suggests that CSCs can exist as two interchangeable populations and Biddle, et al.^3^ (2011) confirmed that CSCs form a dynamic cell population that uses EMT and MET to switch backwards and forwards between a proliferative epithelial phenotype (EPI-CSC; CD44^high^ESA^low/+^ALDH^+^) and a migratory mesenchymal phenotype (EMT-CSC; CD44^high^ESA^low/^, ALDH^-^) (Figure 3). Of particular interest, EMT not only enables cell migration but also alters drug sensitivities so that EPI-CSCs and EMT CSCs respond quite differently to chemo- and radio-therapies^2^ ^,^ ^3^ ^,^ ^16^.

### Future treatment perspectives

Surgical resection is still a major therapy for OSCC and is effective, especially in treating smaller lesions^30^. Current anti-cancer therapies for more advanced lesions are typically based on radio- and chemo-therapeutic agents that target proliferative cancer cells^27^. However, compared to the bulk of tumour cells, the resistance of EPI-CSC populations to such therapies is greatly enhanced due to their slow cell cycle and their mechanisms for rapid DNA repair and drug exclusion^37^. Consequently, although most non-CSC tumour cells may be eradicated with standard therapies, the therapy resistant CSCs may selectively survive the doses of radio- and chemo­ therapies that are achievable without major damages to the surrounding normal structures. With such partially effective therapies, CSCs can be expected to survive through a process similar to natural selection, and their self-renewal capacity can then enable them to regenerate themselves and stimulate the growth of a new tumour. To avoid such recurrence, therapy therefore needs to employ agents, or combinations of agents, which provide widely effective actions, along with better assays which may allow the effective screening of new and existing drugs for their differential effects on all sub-types of CSCs and non-CSCs.

The molecular advances in tumour biology studies are guiding an individualized treatment approach. For example, a clinically validated chemo-predictive assay (ChemoID^®^) is now being tested for HNSCC, in which both CSCs and bulk tumour cells are challenged by various FDA-approved drugs and their combinations to determine the most effective chemotherapy scheme. This assay, although still not FDA-approved, was recently published as a new complementary procedure to HNSCC drug treatment, aiming at both the elimination of unnecessary toxicity in patients as well as avoiding ineffective chemotherapy regimens^10^.

A better understanding of CSC properties is crucial for the development of effective alternative strategies, for example, targeting stem cell maintenance, signalling pathways or blocking EMT/MET to prevent the switching of CSCs between drug resistant phenotypes.
